# A high-resolution daily global dataset of statistically downscaled CMIP6 models for climate impact analyses

**DOI:** 10.1038/s41597-023-02528-x

**Published:** 2023-09-11

**Authors:** Solomon Gebrechorkos, Julian Leyland, Louise Slater, Michel Wortmann, Philip J. Ashworth, Georgina L. Bennett, Richard Boothroyd, Hannah Cloke, Pauline Delorme, Helen Griffith, Richard Hardy, Laurence Hawker, Stuart McLelland, Jeffrey Neal, Andrew Nicholas, Andrew J. Tatem, Ellie Vahidi, Daniel R. Parsons, Stephen E. Darby

**Affiliations:** 1 School of Geography and Environmental Science, University of Southampton, Southampton, SO17 1BJ UK; 2 School of Geography and the Environment, University of Oxford, Oxford, UK; 3 School of Applied Sciences, University of Brighton, Sussex, BN2 4AT Brighton, UK; 4 Department of Geography, Faculty of Environment, Science and Economy, University of Exeter, Exeter, EX4 4RJ UK; 5 School of Geographical & Earth Sciences, University of Glasgow, Glasgow, UK; 6 Geography and Environmental Science, University of Reading, Reading, UK; 7 Energy and Environment Institute, University of Hull, Hull, UK; 8 Department of Geography, Durham University, Lower Mountjoy, South Road, Durham, DH1 3LE UK; 9 School of Geographical Sciences, University of Bristol, Bristol, BS8 1SS UK

**Keywords:** Projection and prediction, Climate and Earth system modelling

## Abstract

A large number of historical simulations and future climate projections are available from Global Climate Models, but these are typically of coarse resolution, which limits their effectiveness for assessing local scale changes in climate and attendant impacts. Here, we use a novel statistical downscaling model capable of replicating extreme events, the Bias Correction Constructed Analogues with Quantile mapping reordering (BCCAQ), to downscale daily precipitation, air-temperature, maximum and minimum temperature, wind speed, air pressure, and relative humidity from 18 GCMs from the Coupled Model Intercomparison Project Phase 6 (CMIP6). BCCAQ is calibrated using high-resolution reference datasets and showed a good performance in removing bias from GCMs and reproducing extreme events. The globally downscaled data are available at the Centre for Environmental Data Analysis (10.5285/c107618f1db34801bb88a1e927b82317) for the historical (1981–2014) and future (2015–2100) periods at 0.25° resolution and at daily time step across three Shared Socioeconomic Pathways (SSP2-4.5, SSP5-3.4-OS and SSP5-8.5). This new climate dataset will be useful for assessing future changes and variability in climate and for driving high-resolution impact assessment models.

## Background & Summary

A large number of climate projections are available from Global Climate Models (GCMs), but these projections are typically of relatively coarse spatial resolution (~1–3°) and with large biases and uncertainties. These GCM data are used to understand and assess potential changes and variability in climate and climate extremes at a global scale^1–3^, but their coarse resolution means that they are not suitable for direct use in impact assessment studies or for decision-making processes at a local scale^4–7^. In addition, GCMs are known to have large biases and uncertainties in representing the historical and future climate, especially for extreme events, and these biases and uncertainties increase from the global to the local scale^8–10^. Overall, the coarse spatial resolution and large bias and uncertainty in GCMs currently limit their applicability for local-scale climate studies which are most meaningful for impact assessments^4,5,10^. Therefore, robust climate data with a high spatial and temporal resolution are urgently needed to assess the impacts of climate change on critical sectors such as agriculture, water resources and energy^4,5,11^.

To develop high-resolution climate data from GCMs and to reduce biases, a number of statistical and dynamical downscaling techniques have been developed^12,13^. Regional Climate Models (RCMs) are dynamical models which use local information such as topography to produce high-resolution (e.g. the Coordinated Regional climate Downscaling Experiment; CORDEX^14^) climate data from GCMs. However, RCMs suffer from large biases, errors and sensitivity to the boundary conditions of the driving GCMs, which limits their application for local scale impact assessments^15,16^. In addition, dynamical models are computationally expensive and require large data storage and processing times^13,15–18^. In contrast, downscaling based on statistical methods provides a high resolution equivalent to downscaling based on dynamical methods, but with much less resource and computational demand^1,19^. Statistical downscaling models are known to significantly reduce biases in individual GCMs and the ensemble means of multiple GCMs at a local scale^6^. Statistical methods involve the development of a statistical relationship between observed and model data during a historical period (e.g., 1981–2014) and then application of this relationship to downscale and bias correct the future climate parameters. In these statistical methods, it is assumed that the established historical link between local scale and large-scale climate variables will remain relatively constant in the future period^13,19^. In general, considering the simplicity and computational advantages of statistical methods they are widely used in climate change and variability, hydro-climate extremes and impact assessment studies at regional and local scales in sectors such as agriculture, energy and water resources^16,20–24^.

During the last few decades, several statistical downscaling methods such as the Bias Correction Constructed Analogues with Quantile mapping reordering (BCCAQ)^25,26^, Quantile Delta Mapping (QDM)^25^, Statistical Downscaling Model (SDSM)^19^, bias correction spatial disaggregation (BCSD)^27^, climate imprint delta method (CI)^28^, bias-corrected climate imprint delta method (BCCI)^28^, and equidistant cumulative distribution function (EDCDF)^29^ have been introduced and used in impact studies. Here, we used the BCCAQ gridded statistical downscaling method to develop daily high-resolution climate datasets globally from 18 CMIP6 (Coupled Model Intercomparison Project Phase 6) models across three Shared Socioeconomic Pathways (SSPs) scenarios. Compared to other gridded downscaling techniques such BCSD, CI, and BCCI, BCCAQ has been demonstrated to have superior performance when the downscaled variables are used for simulating hydrological extremes^26^. BCCAQ has been used to develop high-resolution climate datasets for assessing climate extremes in British Columbia^30^, and climate change impact assessment studies^31,32^ but it has not previously been applied globally. In addition, our new dataset^33^ provides high-resolution data for seven frequently used variables (Table 1) downscaled from 18 GCMs and 3 scenarios, compared with ClimateImpactLab/downscaleCMIP6^34^ which provides only temperature and precipitation datasets based on the Quantile Delta Mapping (QDM) method. Similarly, the NASA global downscaled projection^35^ uses the BCSD method and does not include air pressure, which is required in most hydrological models^36,37^. The Inter-Sectoral Impact Model Intercomparison Project (ISIMIP, https://www.isimip.org/), has also developed downscaled and bias-corrected climate data from CMIP6 models but it has a relatively coarse spatial resolution (0.5°). Our high-resolution (0.25°) daily climate dataset will be useful for assessing changes and variability in the climate and for driving a range of impact assessment models, including hydrological models incorporating analysis of extreme events. The new dataset is freely available to download from the Centre for Environmental Data Analysis (CEDA; 10.5285/c107618f1db34801bb88a1e927b82317)^33^.

**Table 1:** Selected and downscaled climatological variables for the historical (1981–2014) and future (2015–2100) periods.

Variables	Acronym	Units
Precipitation	pr	mm/day
Near-surface (2 meter) air temperature	tas	°C
Maximum near-surface (2 meter) temperature	tasmax	°C
Minimum near-surface (2 meter) temperature	tasmin	°C
Surface air pressure	ps	kPa
Near-surface relative humidity	hurs	%
Surface (10 meter) wind speed	sfcWind	m/s

## Methods

### Data acquisition

Gridded high-resolution bias-corrected meteorological datasets were obtained from GloH2O (http://www.gloh2o.org/mswx/) to calibrate the downscaling model over the historical period (1981–2014). GloH2O provides daily and sub-daily meteorological datasets (Multi-Source Weather; MSWX) such as mean temperature, maximum and minimum temperature, surface pressure, relative humidity and wind speed at a spatial resolution of 0.1° and for the period 1979-present^38^. The MSWX is developed based on multiple observational data sources, downscaling and bias-correction methods. For example, the average air temperature is developed by resampling the Climatologies at High resolution for the Earth’s Land Surface Areas (CHELSA)^39^ dataset to 0.1° and it is corrected using the climatology of the Climatic Research Unit Time Series (CRU TS) data^38^. For precipitation, Multi-Source Weighted-Ensemble Precipitation (MSWEP) available from GloH2O (www.gloh2o.org/mswep) is used. MSWEP is developed by blending multiple sources such as ground observations, satellite and reanalysis datasets^40,41^ and has been shown to better represent extreme events^42^. MSWEP includes more than 77,000 gauge data from Global Historical Climatology Network Daily (GHCNd), Global Summary of the Day (GSOD), and Global Precipitation Climatology Centre (GPCC), remote sensing-based precipitation products such as Climate Prediction Center morphing technique (CMORPH), Tropical Rainfall Measuring Mission (TRMM), Multi-satellite Precipitation Analysis (TMPA), and Global Satellite Mapping of Precipitation (GSMaP), and reanalysis data from the Japanese 55-year reanalysis and European Centre for Medium-Range Weather Forecasts (ECMWF) interim reanalysis. The spatial resolution of MSWEP and MSWX is bilinearly interpolated to 0.25° for the downscaling process.

Herein we chose to assess three future (2015–2100) projections of climate based on the latest Shared Socioeconomic Pathway (SSP) scenarios outlined in the IPCC sixth assessment^43^. SSP2-4.5 represents a commonly used lower bound of warming, whereby a ‘middle of the road’ SSP is selected, keeping CO2 relatively low. In contrast, SSP5-8.5 represents a high-emissions SSP which is reliant upon fossil fuels^44^, but is now considered as unlikely^45^. In addition, we also chose to downscale SSP5-3.4-OS, which is known as an ‘overshoot scenario’, where warming follows a worst-case trajectory until 2040 before a rapid decrease driven by mitigation^44^. The historical and future climate data from the CMIP6 models were obtained from the Centre for Environmental Data Analysis (CEDA, https://esgf-index1.ceda.ac.uk/projects/cmip6-ceda/). We selected 18 GCMs based on the availability of daily data for precipitation, temperature, maximum and minimum temperature, air pressure, relative humidity and wind speed (Table 1). These variables are selected due to their frequent use in many environmental impact assessment models, notably hydrological models^36,37^.

### Downscaling process

The statistical downscaling model used here to develop high-resolution climate data globally is the Bias Correction Constructed Analogues with Quantile mapping reordering (BCCAQ^25,26^). This is a hybrid downscaling model which combines the Bias Correction Constructed Analogs (BCCA^31^) and Bias Correction Climate Imprint (BCCI^28^) to produce daily climate variables, replicating extreme events and spatial covariance effectively^26^. BCCAQ, which combines different downscaling techniques, is more effective in replicating extreme events, spatial covariance and daily sequencing than using a single method^30^. The BCCI method interpolates the coarser climate data from climate models into a finer resolution and bias corrects the data using Quantile Delta Mapping (QDM^25^). The BCCA is used to perform quantile mapping between the climate model data and spatially aggregated reference dataset to the resolution of the climate models. The relationship between the reference dataset and climate model is used to bias-correct the model data. During the downscaling, the BCCI, BCCA and QDM algorithms run independently and the BCCAQ combines the outputs. In previous applications, BCCAQ was used by the Pacific Climate Impacts Consortium to downscale GCM data for Canada (https://data.pacificclimate.org/portal/downscaled_cmip6/map/). Here we apply the technique to global datasets for the first time. BCCAQ is calibrated using reference datasets of precipitation (MSWEP) and weather (MSWX) during the historical period (1981–2014) and then the calibration is used to downscale future scenarios. Further information about the BCCAQ downscaling model can be found at the Pacific Climate Impacts Consortium (PCIC, https://pacificclimate.org/).

Compared to other downscaling methods, such as Statistical DownScaling Model (SDSM) and Bias Correction and Spatial Downscaling (BCSD), BCCAQ is an extremely computationally intensive algorithm, requiring high memory compute nodes (~3 TB RAM per compute node) for global scale downscaling. We used the UK’s data analysis facility for environmental science (JASMIN, https://jasmin.ac.uk/) and the University of Southampton (https://www.southampton.ac.uk/isolutions/staff/iridis.page) and University of Oxford (https://www.arc.ox.ac.uk/home) High-Performance Computing (HPC) resources. The downscaling was implemented using the ClimDown package, written in R (https://github.com/pacificclimate/ClimDown)^46^. Global input data for each of our simulations needed to be divided into 17 smaller areas to enable the analysis to complete within the allocated wall time of the HPC facilities (~48 hrs). The Climate Data Operators (CDO^47^) package was used to split and merge the datasets and adjust model grid types.

### Evaluation methods

To assess the quality of the downscaled data several statistical and graphical methods are used. The downscaling data is compared against the reference dataset using the Pearson correlation coefficient, root mean square error (RMSE), bias and standard deviation. In addition, the Taylor diagram^48^ is used to summarise the performance of individual models for each variable. The Taylor diagram is a graphical method frequently used for comparing a set of variables from observations and models using correlation coefficient, standard deviation, and centred RMSE. Furthermore, extreme indices are used to assess the performance of the downscaled data for detecting extreme events such as heavy precipitation days and very warm and cold days. The indices are based on the definition of the Expert Team on Climate Change Detection and Indices (ETCCDI)^49^. Heavy precipitation is defined as the number of precipitation days where daily precipitation is greater than 10 mm. The very warm days indicate the percentage of days where the daily maximum temperature is greater than the 90^th^ percentile of the daily maximum temperature of the reference period (1981–2014). In addition, very cold days represent the percentage of days where the daily maximum temperature is less than the 10^th^ percentile of the daily maximum temperature of the reference period.

## Data Records

The downscaled (0.25°) daily data from the 18 GCMs for each of the seven climatological variables (Table 2) and three SSP scenarios (SSP2-4.5, SSP5-3.4-0S and SSP5-8.5) for the future (2015–2100) and historical (1981–2014) periods are available at the Centre for Environmental Data Analysis (CEDA, 10.5285/c107618f1db34801bb88a1e927b82317)^33^. The CEDA data can be accessed by anyone from anywhere. The data are available in compressed NetCDF format. Individual files (i.e. global time series of a single variable) are large, each in the order of about 30 (historical) to 98 (SSPs) GB for historical and future data, respectively. As such, whilst they can be downloaded individually for any use, for UK based environmental science researchers they are best accessed via the JASMIN HPC cluster (https://jasmin.ac.uk/), which is linked to CEDA and provides direct access to our data using a linux machine (cd to /badc/evoflood/data/Downscaled_CMIP6_Climate_Data/). Data for each variable is located in one of four folders according to the scenario modelled: Historical, SSP2-4.5, SSP5-3.4OS, and SSP5-8.5. For the future period 2015–2100 the file name conventions for all variables and scenarios are set as “Global_*variable*_Downscaled_*Model*_2015–2100_*experiment*.nc”, where “variable” is the name of the downscaled variable (e.g., pr and tas), “Model” is the name of the downscaled GCM, and “experiment” is the future SSP scenario. For the historical period, the relevant records are denoted “Global_*variable*_Downscaled_*Model*_1981–2014.nc”. Note that, unlike SSP2-4.5 and SSP5-8.5, only a few GCMs provide data for SSP5-3.4-OS (Table 2).

**Table 2:** The 18 selected CMIP6 Global Climate Models (GCMs) showing the availability of downscaled daily variables for the Shared Socioeconomic Pathways (SSPs) 2–4.5 and SSP5-8.5.

GCM	Institute and country of origin	pr	tas	tasmax	tasmin	sfcWind	ps	hurs
ACCESS-CM2^56^	Australian Community Climate and Earth System Simulator, Australia	✓*	✓*	✓*	✓*	✓*	✓	✓*
BCC-CSM2-MR^57^	Beijing Climate Center Climate System Model, China	✓	✓	✓	✓	✓	✓	×
CESM2^58^	Community Earth System Model, USA	✓	✓	✓	✓	✓	×	✓
CMCC-CM2-SR5^59^	Centro Euro-Mediterraneo sui Cambiamenti Climatici, Italy	✓	✓	×	×	✓	✓	✓
CMCC-ESM2^59^	Centro Euro-Mediterraneo sui Cambiamenti Climatici, Italy	✓*	✓*	✓*	✓*	✓*	✓	✓*
GFDL-ESM4^60^	NOAA Geophysical Fluid Dynamics Laboratory, USA	✓	✓	✓	✓	✓	✓	✓
HadGEM3-GC31-LL^61^	UK Met Office Hadley Centre, UK	✓	✓	✓	✓	✓	✓	✓
IITM-ESM^62^	Center for Climate Change Research, Indian Institute of Tropical Meteorology Pune, India	✓	✓	×	×	✓	×	✓
INM-CM4-8^63^	Institute of Numerical Mathematics of the Russian Academy of Sciences, Russia	✓	✓	✓	✓	✓	×	✓
INM-CM5-0^63^	Institute of Numerical Mathematics of the Russian Academy of Sciences, Russia	✓	✓	✓	✓	✓	×	✓
IPSL-CM6A-LR^64^	Institut Pierre Simon Laplace, France	✓*	✓*	✓*	✓*	✓*	✓*	✓*
KACE-1-0-G^65^	National Institute of Meteorological Sciences (NIMS) and Korea Meteorological Administration (KMA), Korea	✓	✓	✓	✓	✓	✓	✓
MIROC6^66^	Atmosphere and Ocean Research Institute, University of Tokyo, Japan	✓*	✓*	✓*	✓*	✓	✓	✓*
MIROC-ES2L^66^	Atmosphere and Ocean Research Institute, University of Tokyo, Japan	✓	✓	✓	✓	✓	✓	✓
MPI-ESM1-2-LR^67^	Max Planck Institute for Meteorology, Germany	✓	✓	✓	✓	✓	✓	✓
MRI-ESM2-0^68^	Meteorological Research Institute, Japan	✓*	✓*	✓*	✓*	✓*	✓*	✓*
NorESM2-MM^69^	Norwegian Climate Center, Norway	✓	✓	✓	✓	✓	✓	✓
UKESM1-0-LL^70^	UK Earth System Modelling project, UK	✓*	✓*	✓*	✓*	✓*	✓	✓*

GCMs that have also been forced with the SSP5-3.4-0 S scenario are indicated by an asterisk (*).

## Technical Validation

### Comparison of downscaled and GCMs data

**Fig. 1:**
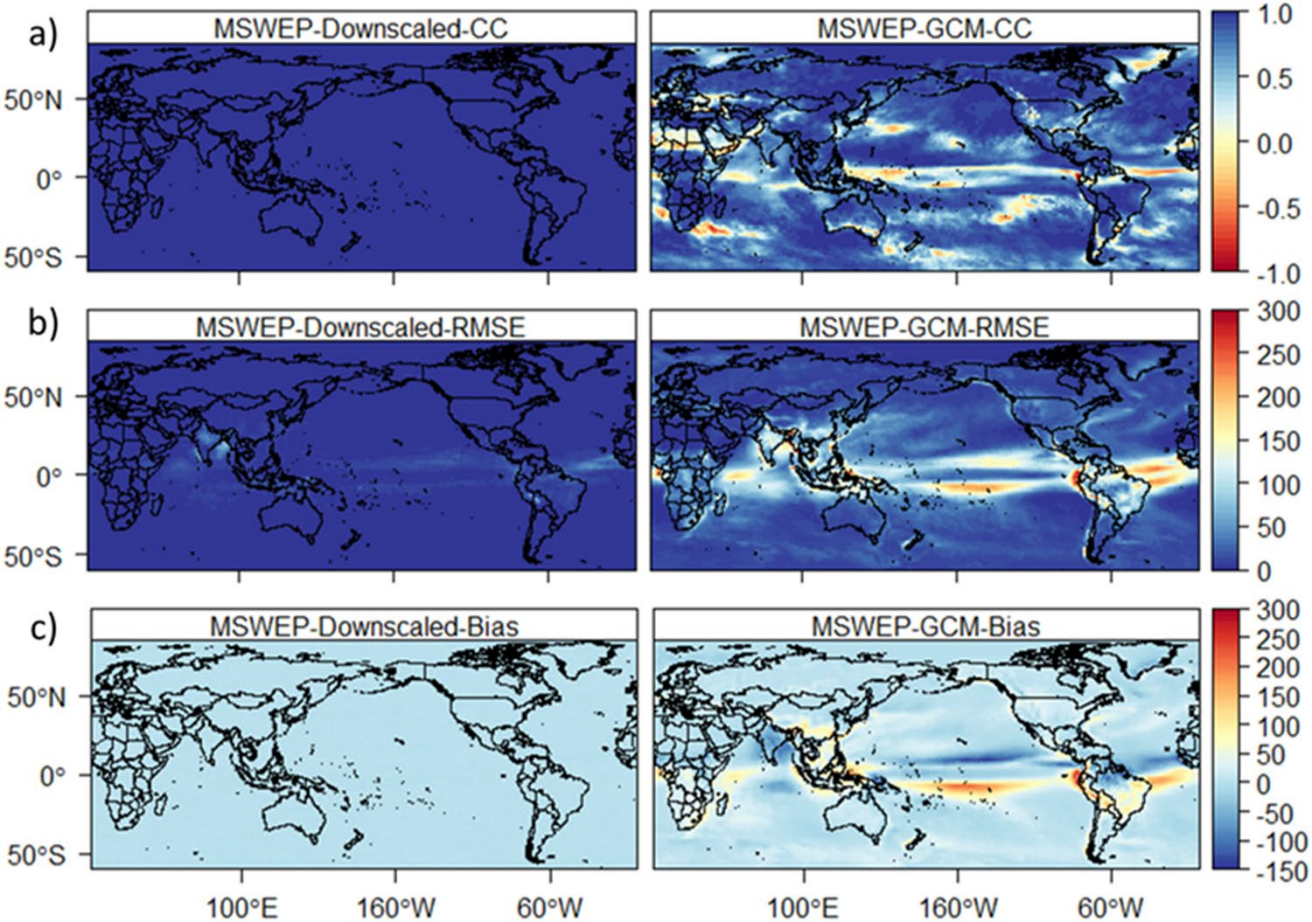
Temporal correlation (**a**), RMSE (**b**) and bias (**c**) between MSWEP and downscaled GCM (ACCESS-CM2, left) and raw GCM (ACCESS-CM2, right) for monthly climatology precipitation during 1981–2014.

**Fig. 2:**
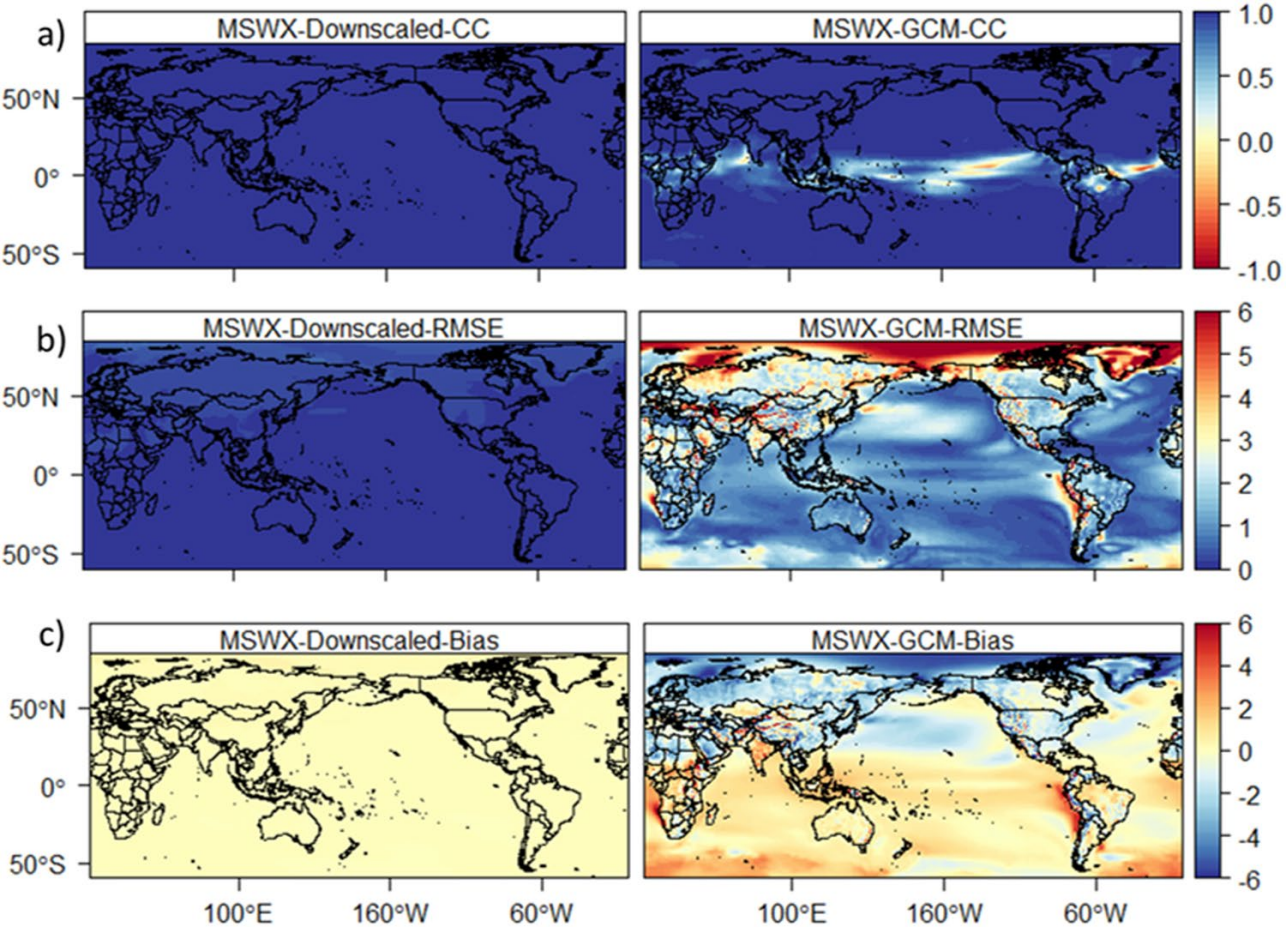
Temporal correlation (**a**), RMSE (**b**) and bias (**c**) between MSWX temperature (MSWX) and downscaled GCM (ACCESS-CM2, left) and raw GCM (ACCESS-CM2, right) for monthly climatological average temperature during 1981–2014.

The downscaled high-resolution datasets are compared with the reference data and raw-GCMs (GCMs) during the period 1981–2014. In addition to producing high-resolution data, the performance of BCCAQ in removing biases and errors in GCMs is assessed. Figure 1 shows a comparison between reference (MSWEP) and downscaled and a GCM (ACCESS-CM2) climatological precipitation (pr). The downscaled data shows a higher correlation and lower bias and RMSE compared to the GCM. On the contrary, the GCM shows a large bias (up to ± 150 mm) and Root Mean Square Error (RMSE) in different parts of the world, particularly in Asia and South America and the Indian and Pacific oceans. The RMSE of monthly climatology precipitation from the GCM is very high compared to the downscaled GCM (Fig. 1b). The downscaled precipitation shows a maximum error (up to 100 mm) only in parts of India. In contrast, the GCM showed an error of up to 300 mm in Africa, Asia and South America. Additionally, the downscaled data show a very low bias compared to the GCM, which showed a bias of up to 300 mm (Fig. 1c). Unlike the high correlation between the downscaled and reference data, the GCM shows a lower correlation in different parts of the world (Fig. 1a). The downscaled climatological average temperature (tas) shows a higher correlation and lower bias and RMSE compared to the GCM (Fig. 2). For example, the GCM shows a lower correlation in Central Africa and South America and a large bias (±5 °C) and RMSE (up to 6 °C) globally. Overall, the GCMs show a large bias and RMSE for all variables compared to the downscaled data.

**Fig. 3:**
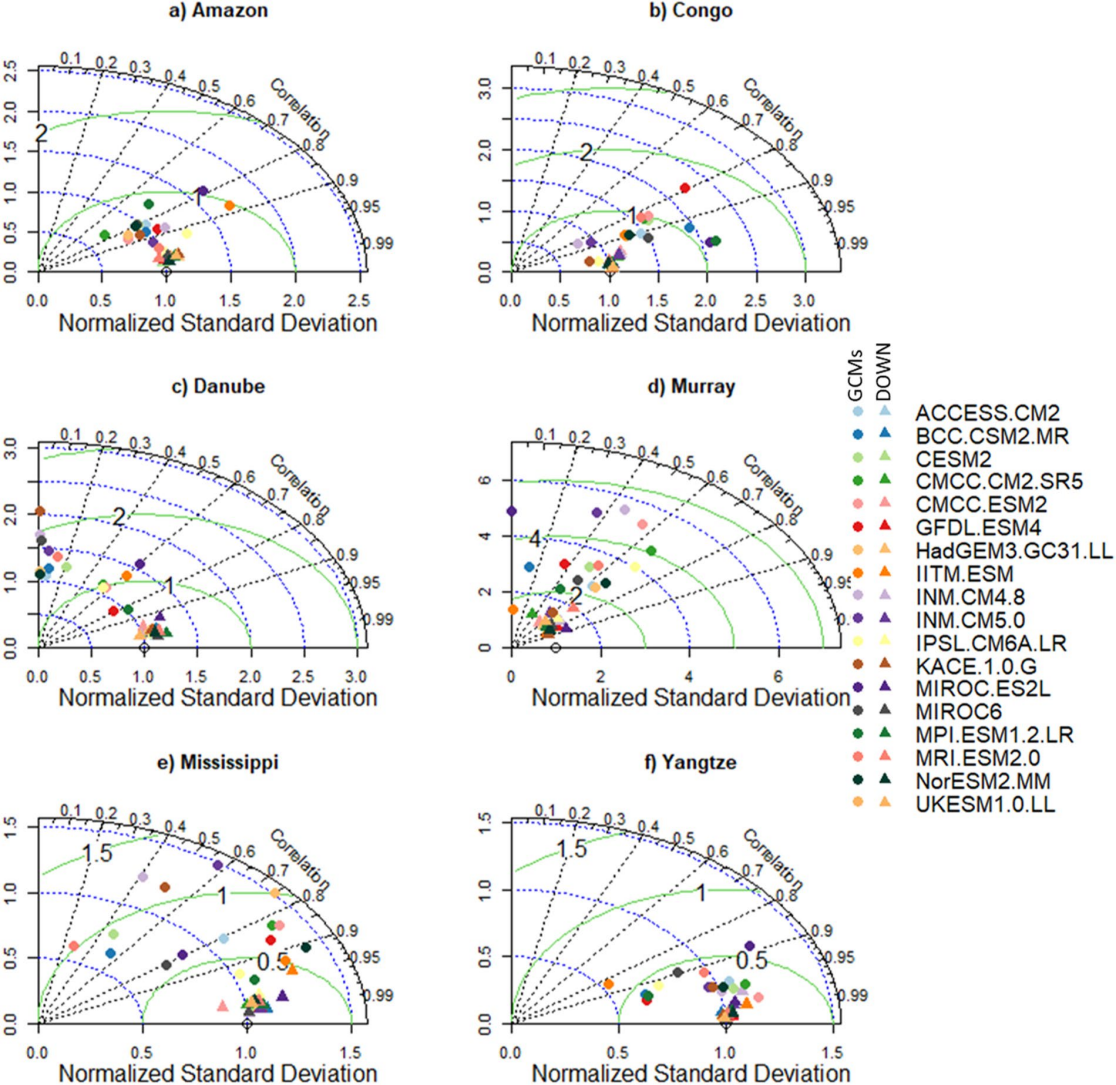
Comparison of Statistically Downscaled (DOWN, triangle) and raw GCMs (GCMs, circles) climatological precipitation for (**a**) Amazon, (**b**) Congo, (**c**) Danube, (**d**) Murray-Darling (Murray), (**e**) Mississippi, and (**f**) Yangtze.

**Fig. 4:**
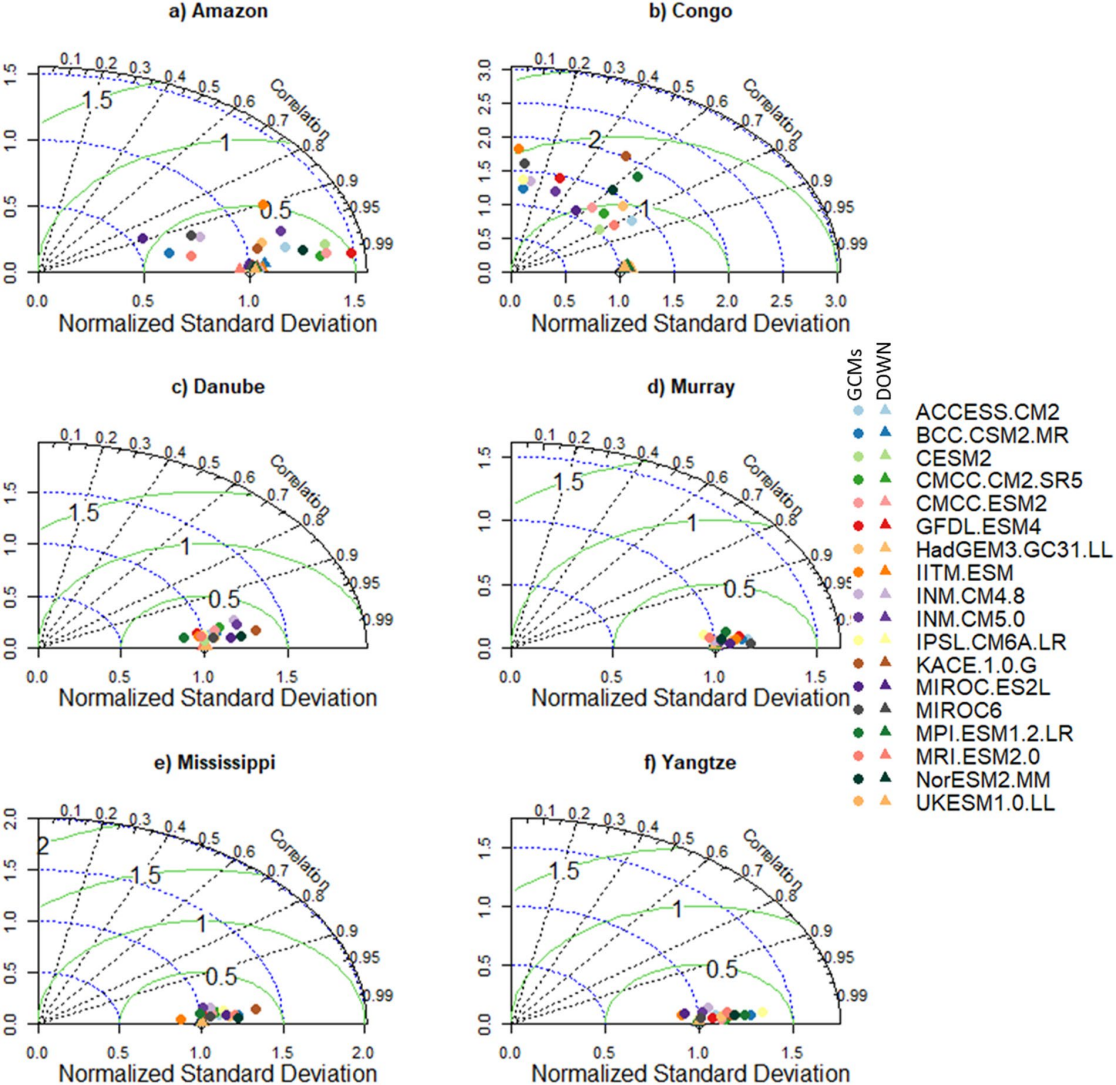
Comparison of Statistically Downscaled (DOWN, triangle) and raw GCMs (GCMs, circles) climatological average temperature for (**a**) Amazon, (**b**) Congo, (**c**) Danube, (**d**) Murray-Darling (Murray), (**e**) Mississippi, and (**f**) Yangtze.

**Fig. 5:**
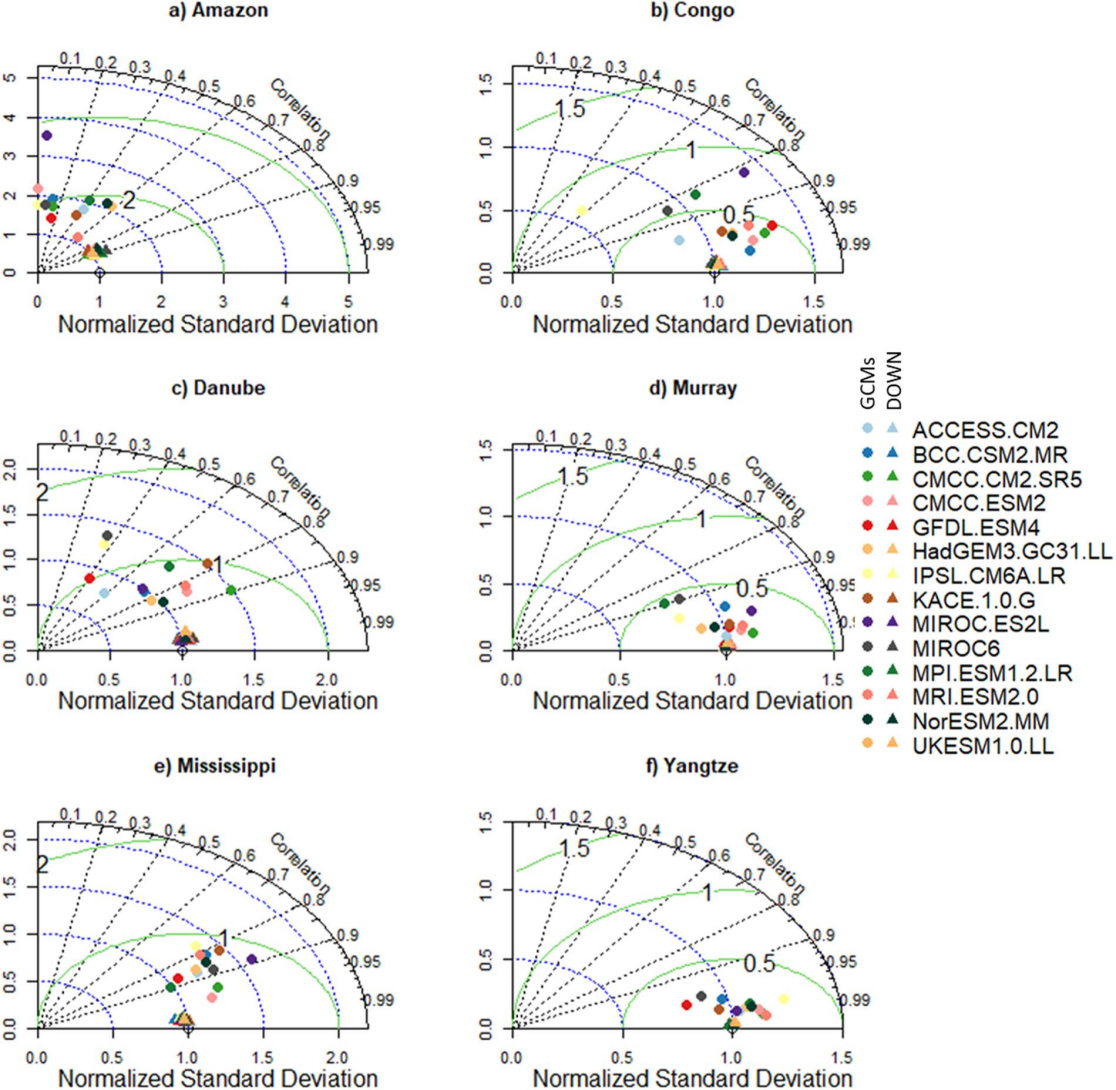
Comparison of Statistically Downscaled (DOWN, triangle) and raw GCMs (GCMs, circles) climatological average surface air pressure (ps) for (**a**) Amazon, (**b**) Congo, (**c**) Danube, (**d**) Murray-Darling (Murray), (**e**) Mississippi, and (**f**) Yangtze.

**Fig. 6:**
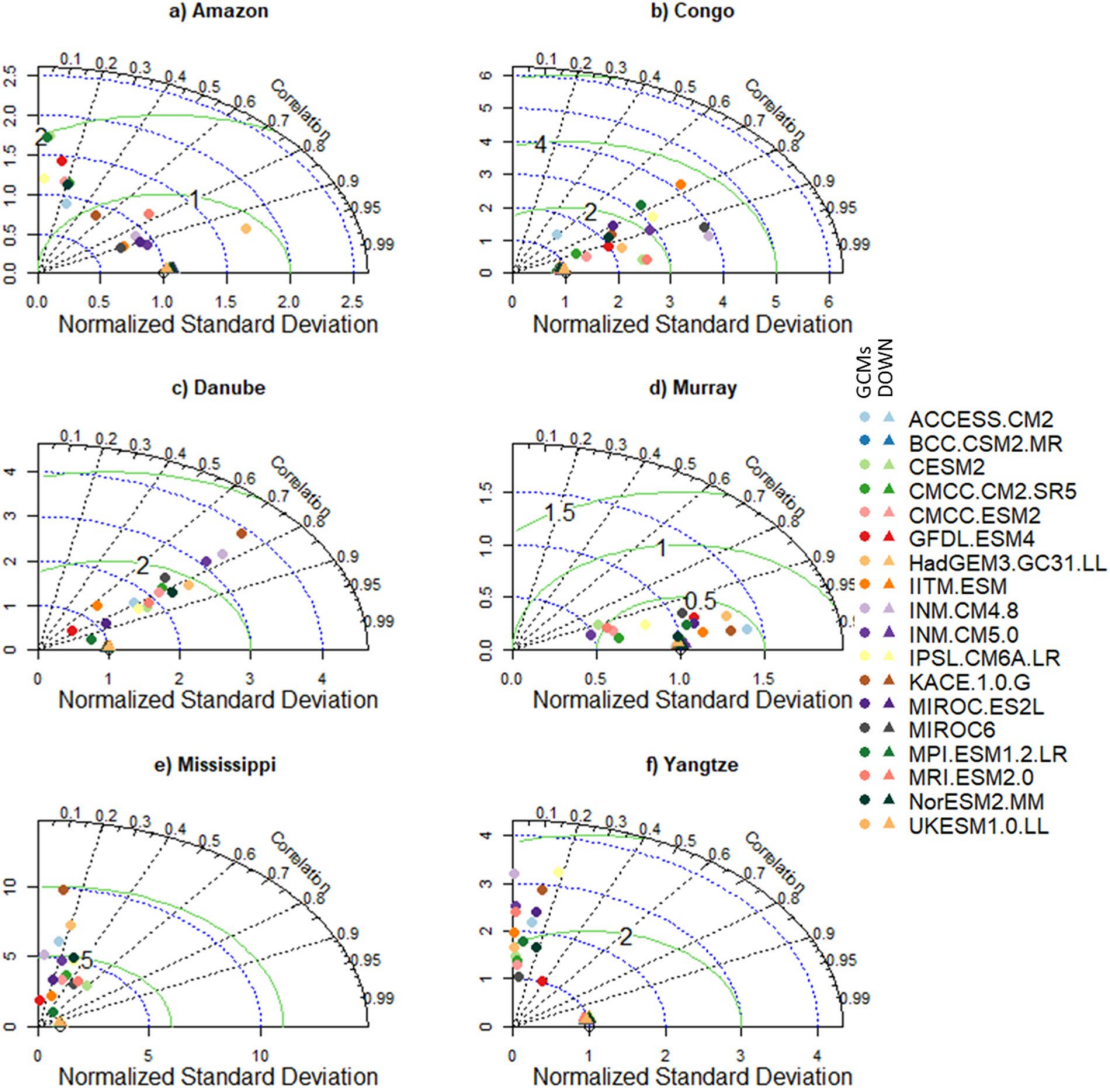
Comparison of Statistically Downscaled (DOWN, triangle) and raw GCMs (GCMs, circles) climatological average relative humidity (hurs) for (**a**) Amazon, (**b**) Congo, (**c**) Danube, (**d**) Murray-Darling (Murray), (**e**) Mississippi, and (**f**) Yangtze.

**Fig. 7:**
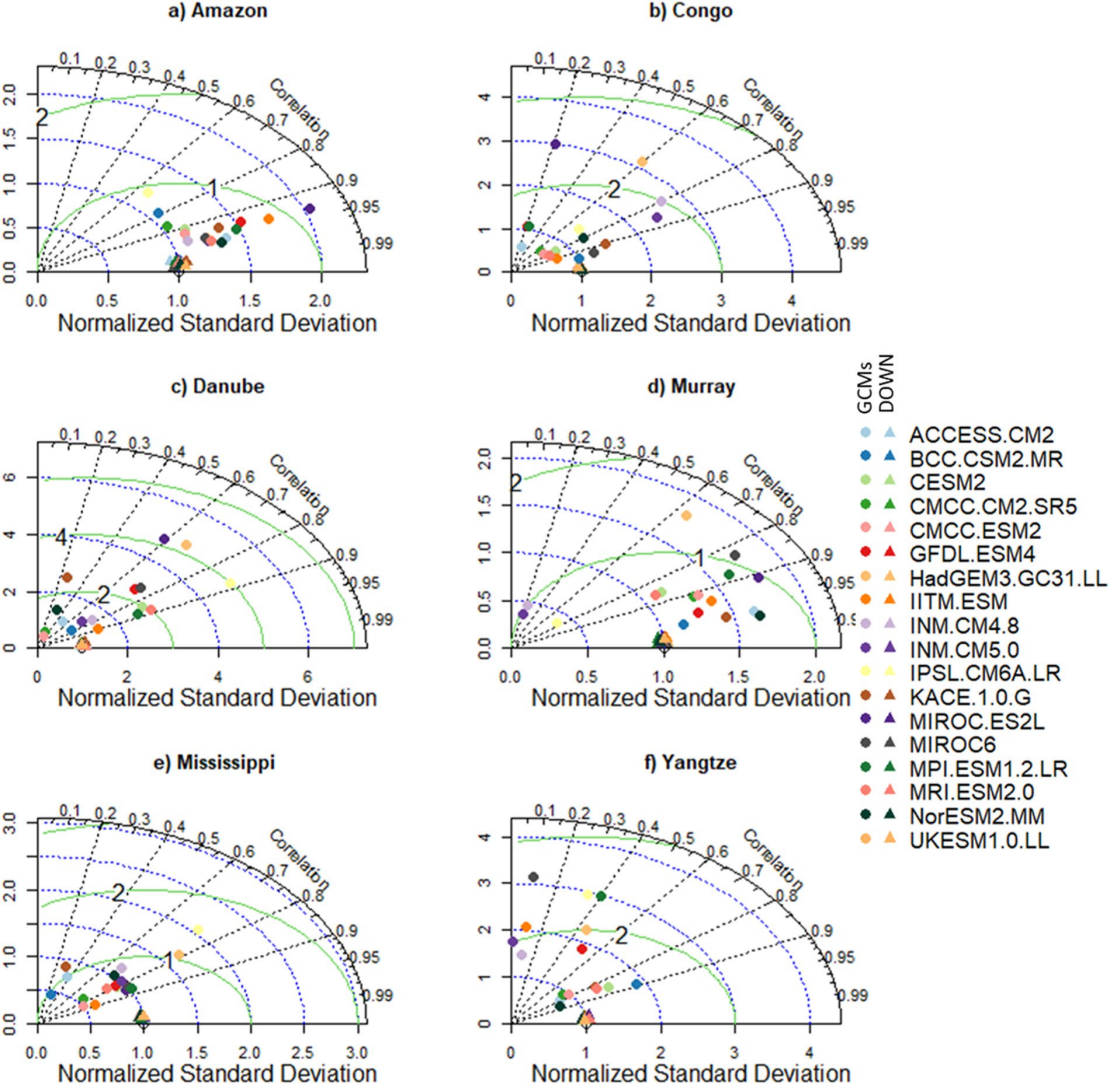
Comparison of Statistically Downscaled (DOWN, triangle) and raw GCMs (GCMs, circles) climatological average wind speed (sfcWind) for (**a**) Amazon, (**b**) Congo, (**c**) Danube, (**d**) Murray-Darling (Murray), (**e**) Mississippi, and (**f**) Yangtze.

**Fig. 8:**
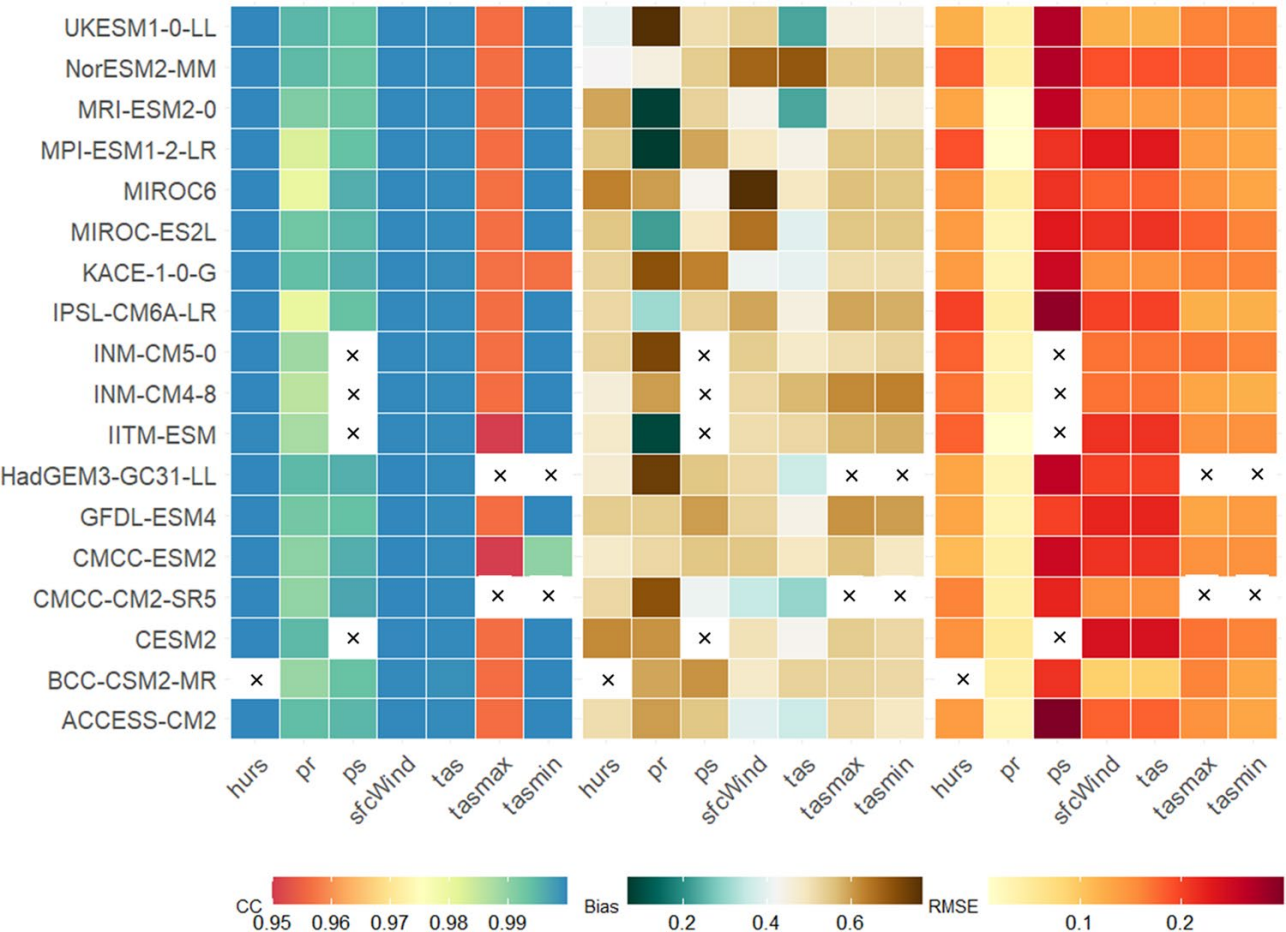
The spatial average correlation (CC), bias, and RMSE between the reference and the downscaled models for climatological average hurs, pr, ps, sfcWind, tas, tasmax, and tasmin. The bias and RMSE are normalised using the maximum and minimum values of the bias and RMSE, respectively. A normalized 0.5 means that the bias or RMSE falls between the minimum and maximum bias and RMSE value in the dataset. The X mark indicates the non-availability of downscaled data.

To highlight the need for bias correlation and spatial downscaling and the utility of our new dataset, we selected six morpho-climatologically diverse river basins from around the world. The basins are Amazon, Congo, Danube, Murray–Darling (Murray), Mississippi, and the Yangtze. For each basin, the climatological average of the seven variables from the downscaled and GCMs are compared against the reference dataset. The comparison between the reference and downscaled and GCMs for all the variables and selected basins is summarised in Figs. 3–8. For climatological average pr, most of the downscaled models show a correlation higher than 0.95 and a similar standard deviation (SD) to the reference datasets (Fig. 3). The GCMs, however, show a lower correlation and a higher SD and centred RMSE than the downscaled data in all the basins. Comparing all the basins, the downscaled data showed a lower correlation (0.4–0.92) in Murray, although this was still considerably better than the GCMs. For tas, the downscaled data, compared to GCMS, show a higher correlation in all the basins (Fig. 4). In addition to the lower correlation, the GCMs show a higher SD and cRMSE than the downscaled data. For example, in Congo, the GCMs show a correlation between 0.1 to 0.8 with a mean of 0.42, whereas the downscale data show a correlation higher than 0.98. The performance of the downscale data is also clear for air pressure (ps, Fig. 5), relative humidity (hurs, Fig. 6), and wind speed (sfcWind, Fig. 7), which show a higher correlation, similar SD to the reference data, and lower centred RMSE. In general, the downscaled data is more accurate than the GCMs in terms of correlation, bias, and errors.

### Global comparison of downscaled and reference datasets

The comparison between the downscaled and GCMs clearly shows the advantage of the downscaling method in removing biases and errors from GCMs and developing high-resolution climate datasets to drive impact models. Here we focus on assessing the performance of the downscaling model in reproducing the climatology of the reference dataset. Figure 8 shows the global average (averaged over all grids) correlation, bias, and RMSE for all the models and variables. Based on the global average correlation, all models perform very well for hurs, sfcWind, tas, and tasmin with a correlation of higher than 0.98. It is, however, slightly lower (>0.95) for pr and tasmax. In addition to the high correlation, the average bias and RMSE are very low for the variables. For example, the average bias and RMSE for tas and pr are 0.06 °C and 0.1 °C and 0.25 mm and 5.1 mm, respectively.

To identify the performance of the downscaled data from all the models and all variables spatial correlation (SFigs. 1–5) and RMSE (SFigs. 6–10) maps are provided in the supplementary material. It is evident from the global maps that the CC is higher than 0.8 for all variables in most parts of the world. This high correlation suggests that the downscaling model has performed well in downscaling the variables and may also represent any biases in the reference dataset. Compared to wind speed (SFig. 2), temperature (SFig. 3) and relative humidity (SFig. 4), which all have a CC of greater than 0.9 in all parts of the world, the correlations obtained are typically slightly lower for precipitation (SFig. 1). For precipitation, the MPI-ESM1-2-LR and IITM-ESM GCMs reveal a lower CC (up to −0.6) in parts of Central Africa, but show similar performance to other downscaled GCMs (typical correlations >0.8) in other parts of the world.

Similarly, the downscaled data show a lower RMSE in most of the world (SFigs. 6–10). For precipitation, the IPSl-CM6A-LR, INM-CM4-8 and INM-CM5-0 show the highest RMSE up to 120 mm in South America (SFig. 6). The downscaled sfcWind data also shows a lower RMSE over land compared to Oceans, which shows an error of up to 0.3 m/s (SFig. 7). The BCC-CSM2-MR, compared to the other models show the highest RMSE over the Arctic Ocean (~0.3 m/s). Most of the downscaled models show a similar pattern of error for tas (up to 0.98 °C), particularly over the temperate zone and the Arctic Ocean (SFig. 8). The average RMSE of the hurs of all models is between 0.2 and 0.4% (SFig. 9). Unlike to the error in sfcWind, hurs show higher RMSE over land compared to Oceans. The BCC-CSM2-MR, IPSL-CM6A-LR, MRI-ESM2-0, and NorESM2-MM, compared to the other models, show a higher RMSE (up to 0.12 kPa) for ps over the Arctic Ocean (SFig. 10). However, most of the models show a smaller RMSE for ps over the land. Overall, the climatology of the downscaled data from all the models and variables shows good agreement with the observed data.

### Time series of global average downscaled and reference datasets

The global average annual pr, sfcWind, tas, hurs, and ps are also well reproduced by the downscaling model (SFigs. 11–15). The global average encompasses both land and ocean areas across all longitudes, spanning latitudes from 60°S to 85°N. Global average pr based on the reference datasets (i.e., MSWEP) during 1981–2010 is 1083 mm with a standard deviation (SD) of 13 mm (SFig. 11). All downscaled models reproduce a similar annual average pr with a SD of between 9.9 mm and 31.3 mm. Even though the models reproduce the global average annual precipitation very well, some models such as CMCC-CM2-SR5, IPSL-CM6A-LR and NorESM2-MM showed a higher annual variability with a SD of about 31 mm. In addition, ACCESS-CM2, CMCC-ESM2, MPI-ESM1-2-LR and MRI-ESM3-0 show a SD of about 21 mm. The multi-model mean (MMM) of all models also shows an average precipitation of 1082.7 mm.

Global average annual tas based on the reference dataset is 16.52 °C (SD = 0.2 °C) and this was well reproduced by all models (between 16.50 °C–16.52 °C) and the MMM (16.51 °C) (SFig. 12). Compared to the other models, BCC-CSM2-MR, CMCC-CM2-SR5, HadGEM3-GC31-LL, IPSL-CM6A-LR, and UKESM1 show a higher annual variability (SD between 0.3–0.34 °C). Similarly, the average annual sfcWind is well reproduced by all the models (SFig. 13). The global average sfcWindfrom the reference data and all models and MMM is 5.99 m/s. Compared to the individual models (SD = 0.3 m/s) and MMM (SD = 0.1 m/s), the reference data show a slightly higher annual variability (SD of 0.6 m/s). The average annual hurs, similar to sfcWind, is accurately reproduced by all the models and MMM (SFig. 14). Based on the reference and all the models, the global average annual hurs is 74.9%. The standard deviation of all the models is 0.1%, whereas the reference datasets show an SD of 0.2%. Further, the downscaled GCMs accurately represent the average annual ps when compared to the reference dataset (SFig. 15). The global average ps from the reference and individual models and MMM is 99.17 kPa and shows a similar (except BCC-CSM2-MR and MRI-ESM2) SD of 0.1 kPa. The BCC-CSM2-MR and MRI-ESM2 show an SD of 0.2 kPa.

### Daily climate extremes

**Fig. 9:**
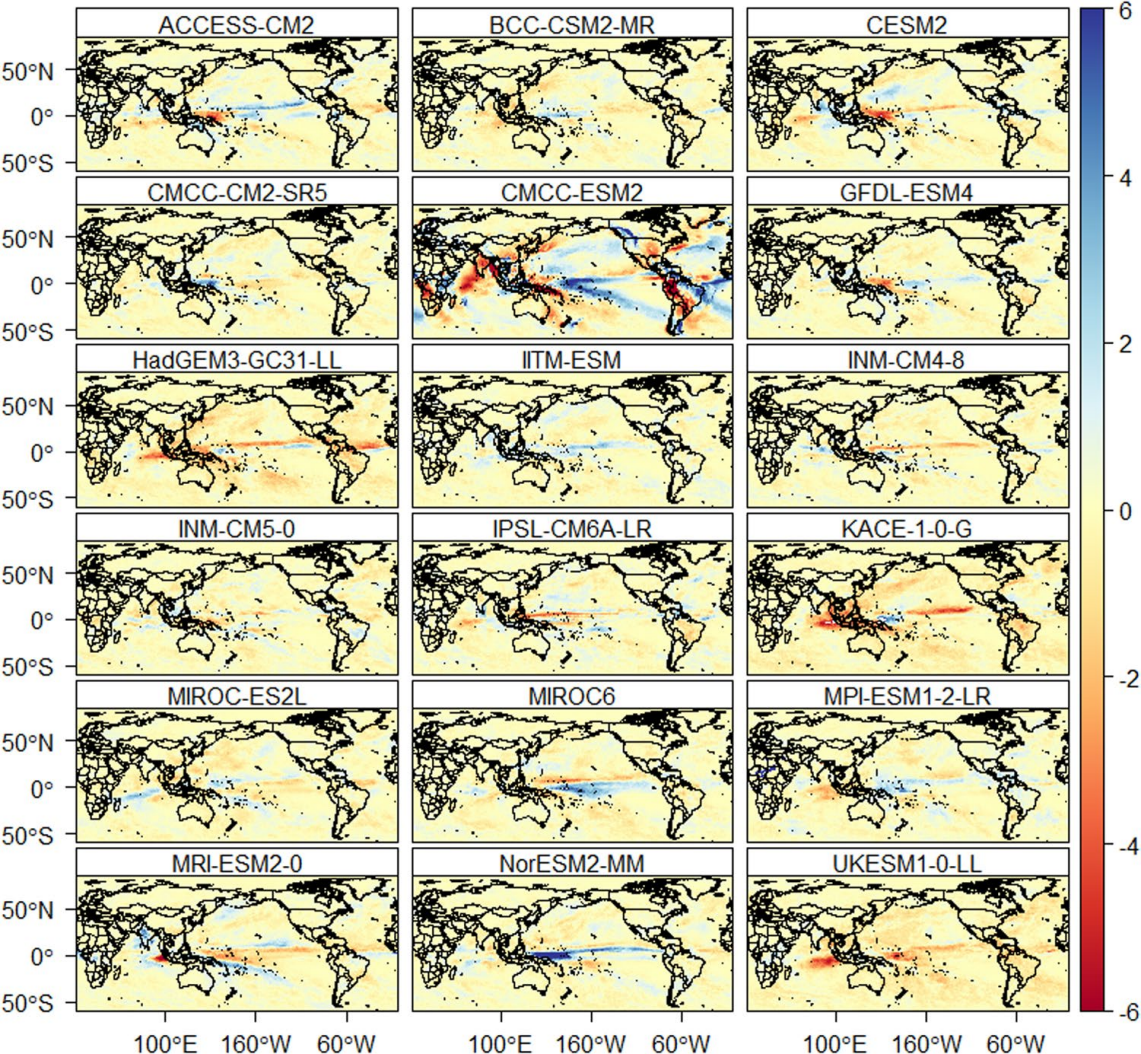
The difference in average annual number of heavy precipitation days (days/year) between the downscaled models and the reference data. The red and blue colour indicates underestimation and overestimation of heavy precipitation days, respectively.

**Fig. 10:**
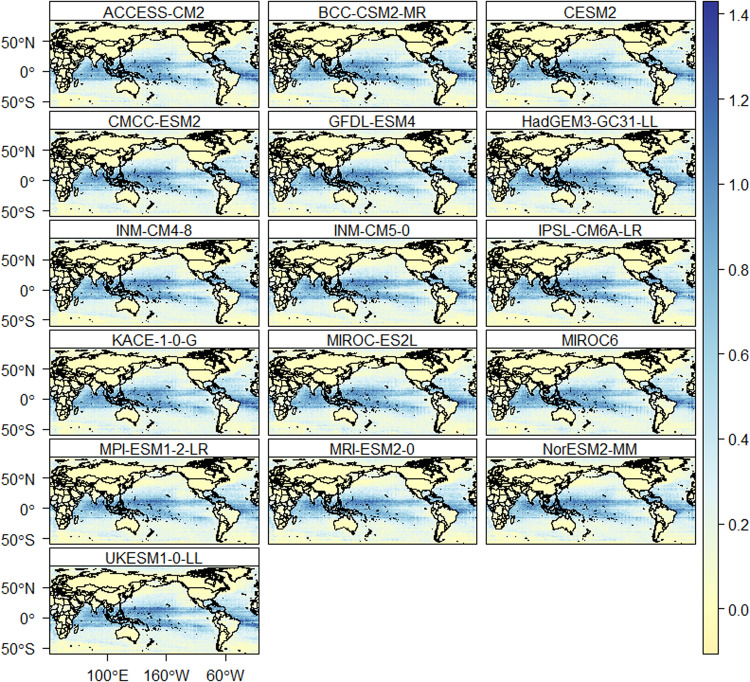
The difference in percentage of very warm days (%) between the downscaled models and the reference data. The blue colour indicates an overestimation of the percentage of very warm days (%).

**Fig. 11:**
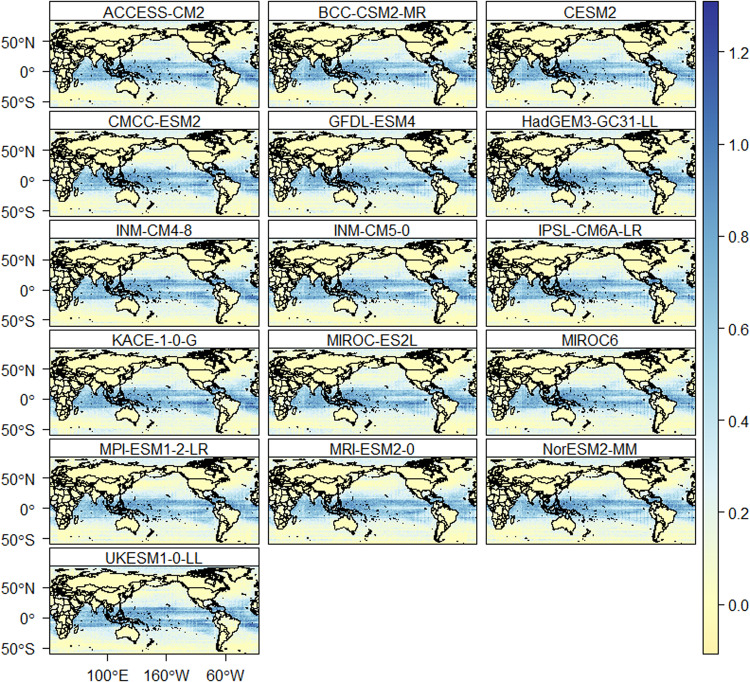
The difference in percentage of very cold days (%) between the downscaled models and reference data. The blue colour indicates an overestimation of the percentage of very cold days (%).

The downscaled data is also assessed for daily extreme events. The number of heavy precipitation days is well reproduced by all models (Fig. 9). Figure 9 provides the average difference in the number of annual heavy precipitation days between the models and reference data. Most of the models show an accurate representation of the number of heavy precipitation days over land compared to oceans. Based on the reference data, the average number of heavy precipitation days is 25 days per year. The CMCC-ESM2, compared to the other models, show a higher difference (±6 days per year) with reference data for heavy precipitation days such as in South America, Asia, and Africa. The average percentage of very warm days based on the reference data is 8.5% of the reference period. All models reproduce the percentage of very warm days very well over the land, except in some places of South East Asia (Indonesia and Thailand) and Central America (Fig. 10). However, all models show a higher percentage of very warm days (up to 1.4% higher than the reference data) over the oceans (Pacific, Indian and Atlantic oceans). Similarly, the average percentage of very cold days based on the reference dataset is 8.5%. All the models represented the percentage of very cold days very well over land than oceans (Fig. 11). The percentage of wet days is overestimated by up to 1.3% in oceans and few areas in South East Asia and Central America.

In summary, the downscaled data accurately reproduces observed data from the historical period for most areas. The high correlation and accurate representation of the global annual and climatological averages of all variables suggest that the downscaling model might also capture any biases in the reference dataset. Even though we used the most comprehensive and high-resolution historical climate datasets to calibrate and downscale the GCMs, it is the case that these datasets might add additional uncertainties in the historical and future climate through propagation of any errors. Specific to precipitation, as a key driver of global hydrological simulations, MSWEP has been evaluated globally and used in various hydro-climate studies^50,51^. Based on recent evaluations^52^, MSWEP was found to outperform 22 other global and quasi-global precipitation datasets such as European Centre for Medium-range Weather Forecasts ReAnalysis Interim (ERA-Interim)^53^, Japanese 55-year ReAnalysis (JRA-55)^54^, and National Centers for Environmental Prediction (NCEP) Climate Forecast system reanalysis (NCEP-CFSR)^55^. In addition, MSWEP was found to capture extreme events better than other satellite-based precipitation datasets^42^. Finally, we note that alongside the uncertainties in the reference climate datasets, it is important to consider the assumptions made in statistical downscaling models (e.g., the assumption of stationarity). However, these uncertainties aside, we are confident that our new downscaled high-resolution climate data can be used in global, regional and local scale impact assessment studies with high accuracy compared to GCMs.

### Supplementary information

Supplementary Information

## Data Availability

The BCCAQ code used to downscale the CMIP6 GCMs can be found at the Pacific Climate Impacts Consortium (PCIC, https://pacificclimate.org/resources/software-library) page and on the R Package Documentation (https://rdrr.io/cran/ClimDown/).
