# Supplementary material for: A high-resolution daily global dataset of statistically downscaled CMIP6 models for climate impact analyses
Source: Sci Data. 2023 Sep 11;10:611. doi: 10.1038/s41597-023-02528-x (PMC10495318; doi:10.1038/s41597-023-02528-x)
Supplement: Supplementary file 1 — Supplementary Information [file 41597_2023_2528_MOESM1_ESM.docx]

### **Supplementary figures**


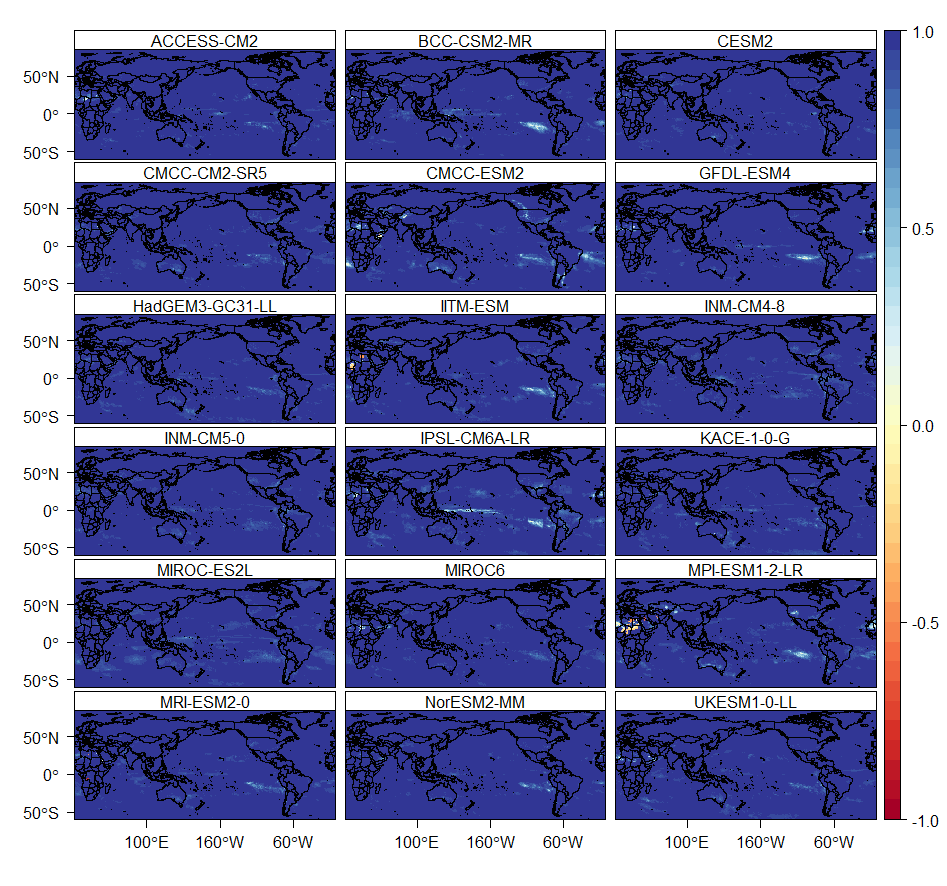


SFig. 1. Temporal correlation of monthly climatological precipitation (pr) between the reference (MSWEP) and downscaled data from 18 GCMs (Table 2) during the period 1981-2014. The correlation is based on the Pearson correlation coefficient.


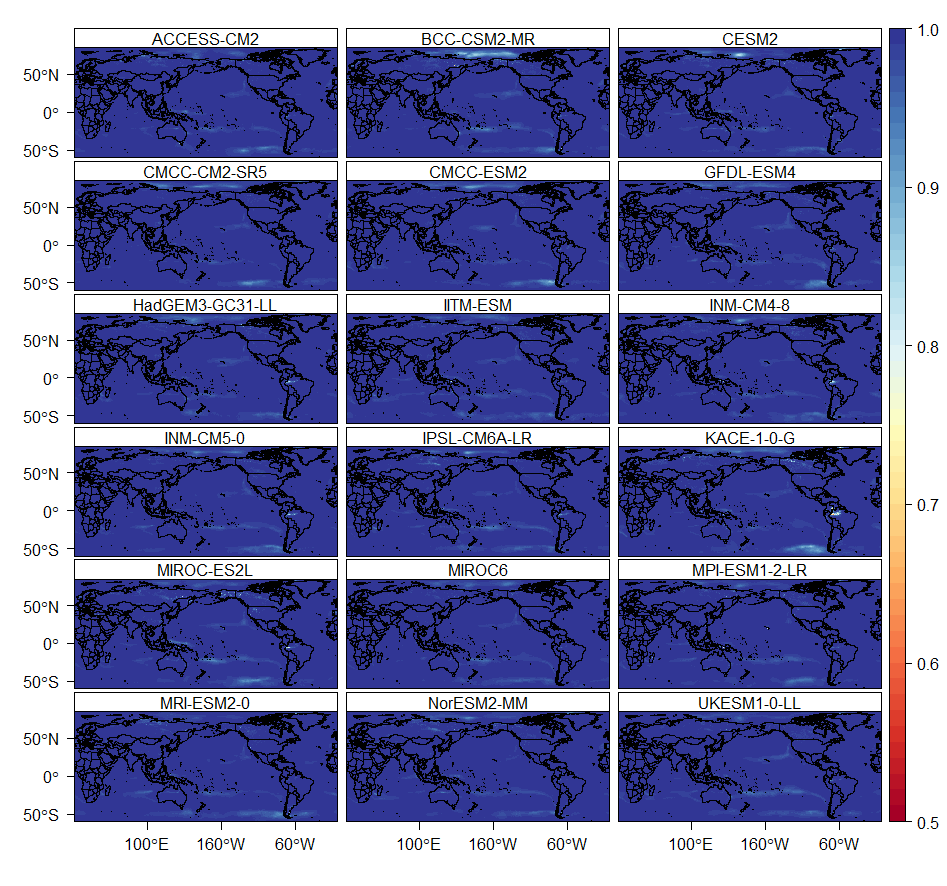


SFig. 2. Temporal correlation of monthly average climatological wind speed (sfcWind) between the reference (MSWX wind speed) and downscaled data from 18 GCMs (Table 2) during the period 1981-2014. The correlation is based on the Pearson correlation coefficient.

**
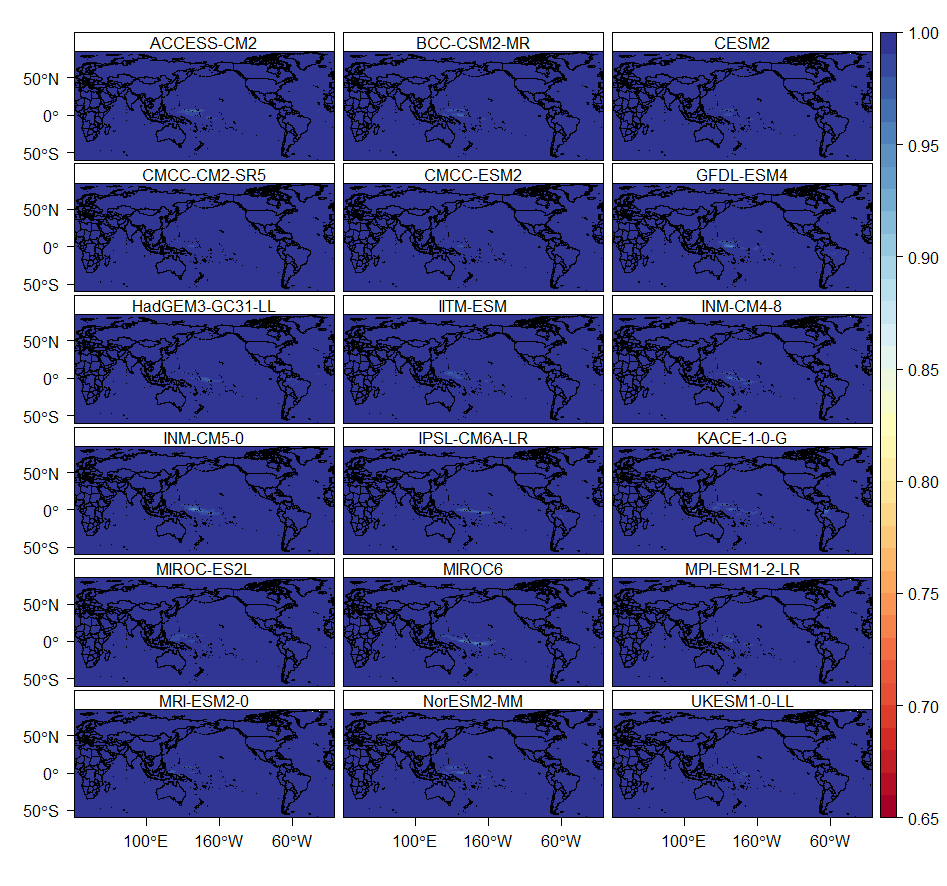
**

SFig. 3. Temporal correlation of monthly climatological average temperature (tas) between the reference (MSWX temperature) and downscaled data from 18 GCMs (Table 2) during the period 1981-2014. The correlation is based on the Pearson correlation coefficient.

**
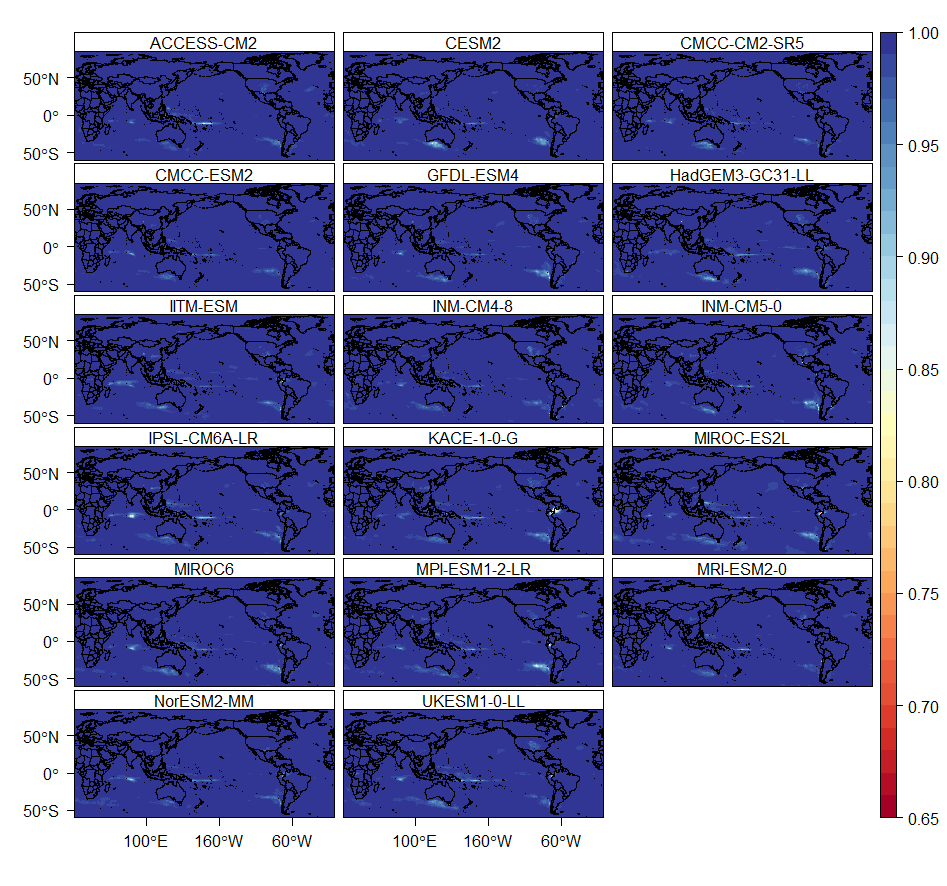
**

SFig. 4. Temporal correlation of monthly climatological relative humidity (hurs) between the reference (MSWX relative humidity) and downscaled data from 17 GCMs (Table 2) during the period 1981-2014. The correlation is based on the Pearson correlation coefficient.


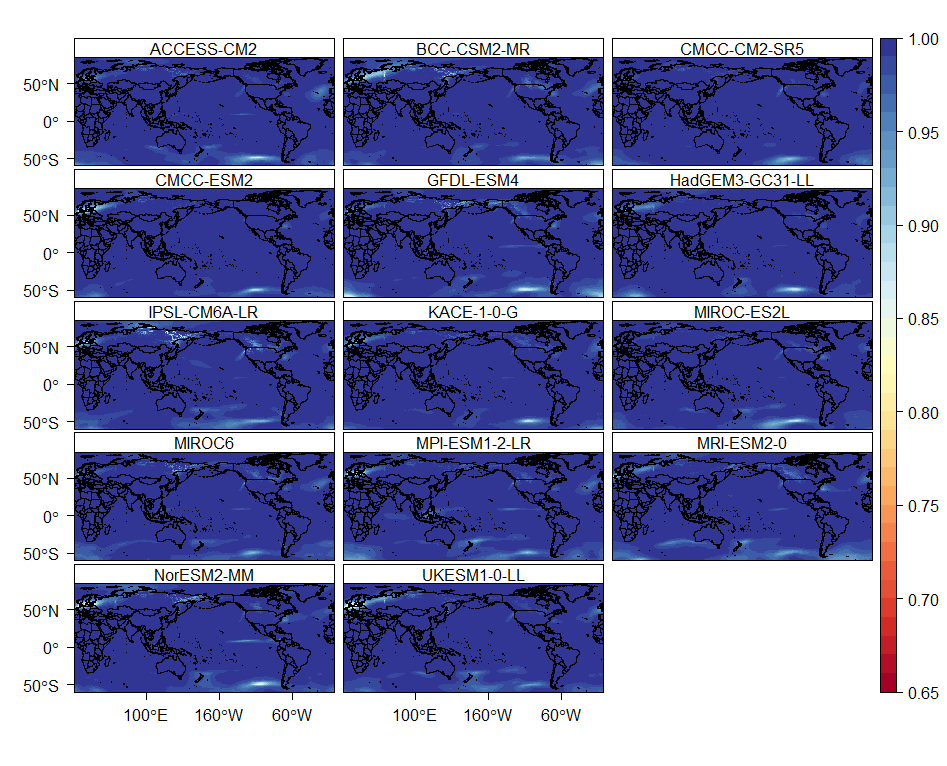


SFig. 5. Temporal correlation of monthly climatological air pressure (ps) between the reference (MSWX air pressure) and downscaled data from 14 GCMs (Table 2) during the period 1981-2014. The correlation is based on the Pearson correlation coefficient.


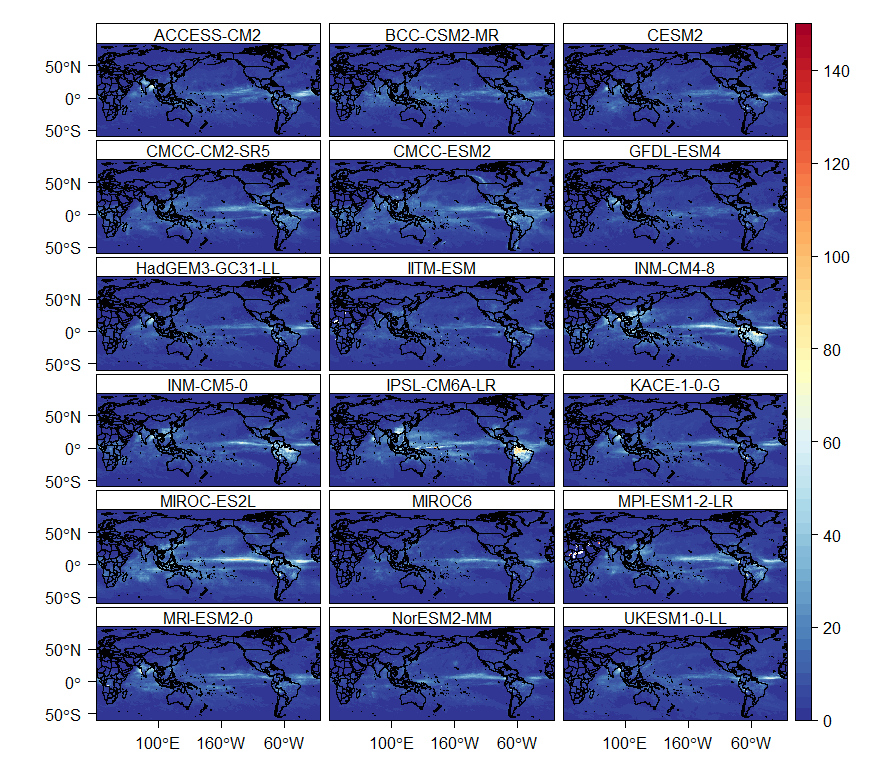


SFig. 6. RMSE of monthly climatological precipitation (pr, mm) between the reference data and all models during 1981-2014.


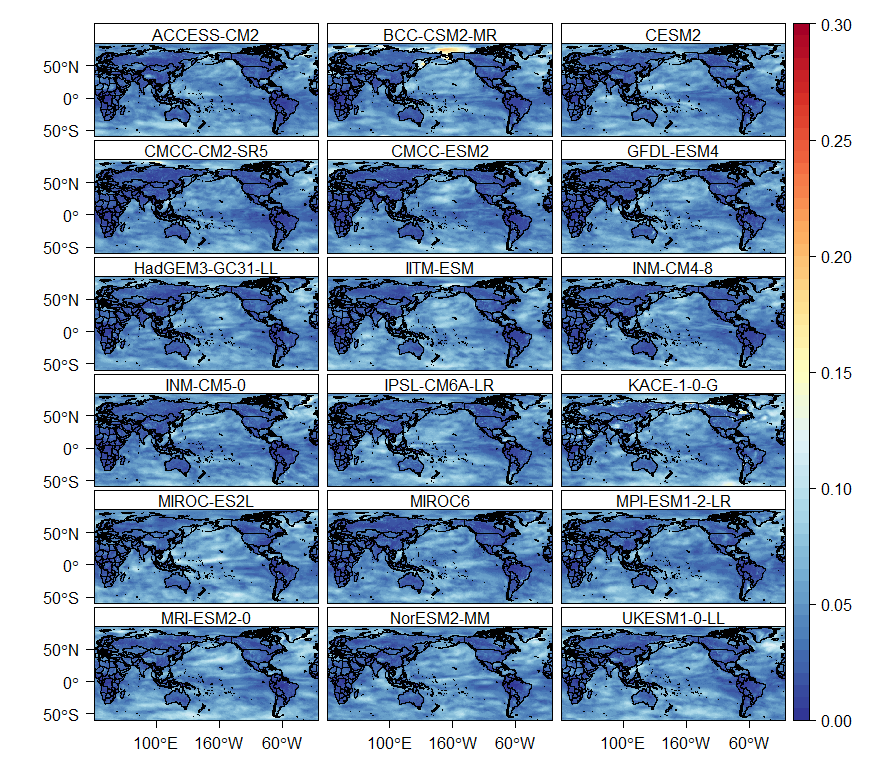


SFig. 7. RMSE of monthly climatological wind speed (sfcWind, m/s) between the reference data and all models during 1981-2014.


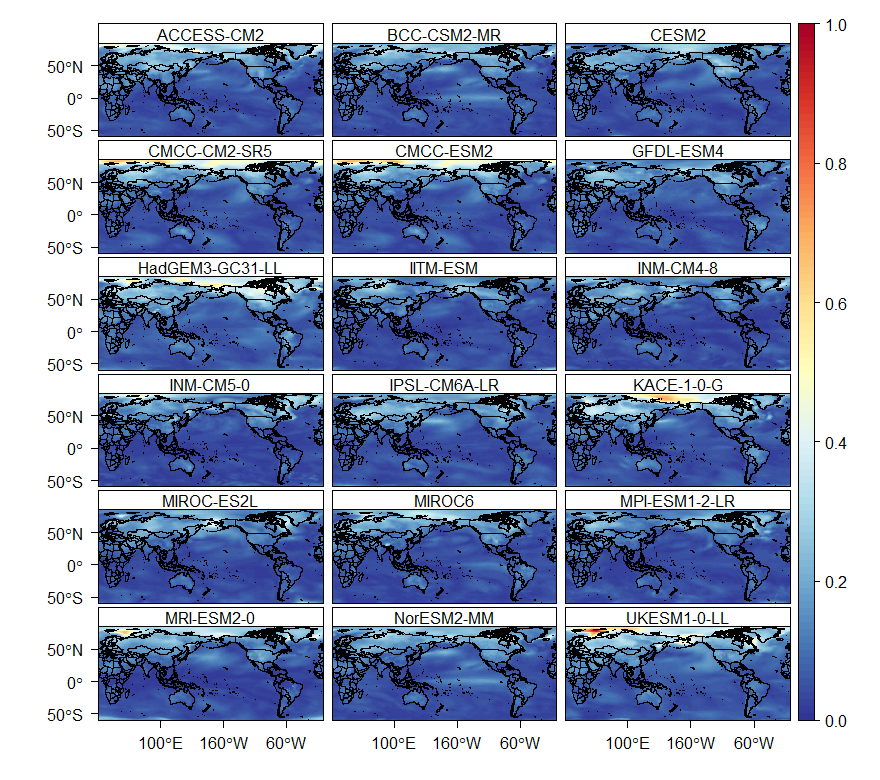


SFig. 8. RMSE of monthly climatological average temperature (tas, °C) between the reference data and all models during 1981-2014.


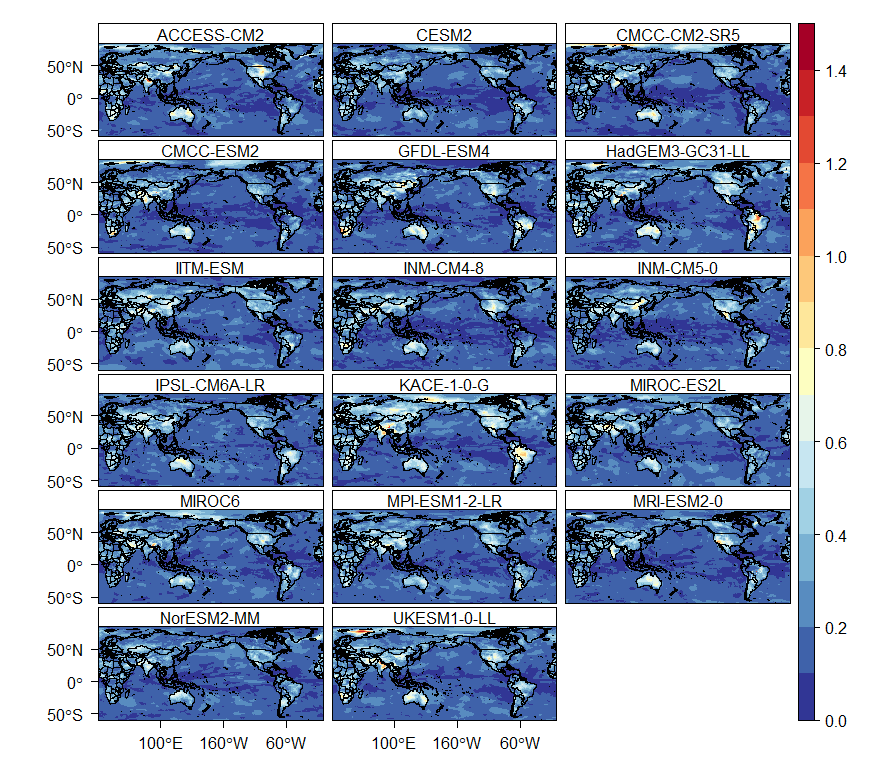


SFig. 9. RMSE of monthly climatological relative humidity (hurs, %) between the reference data and all models during 1981-2014.


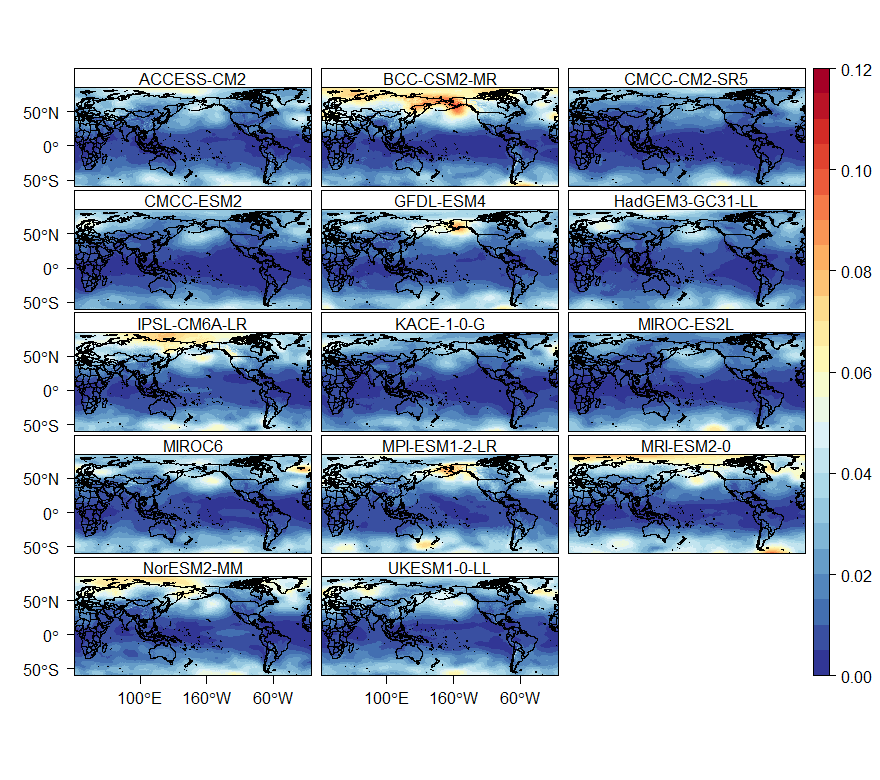


SFig. 10. RMSE of monthly climatological air pressure (ps, kPa) between the reference data and all models during 1981-2014.


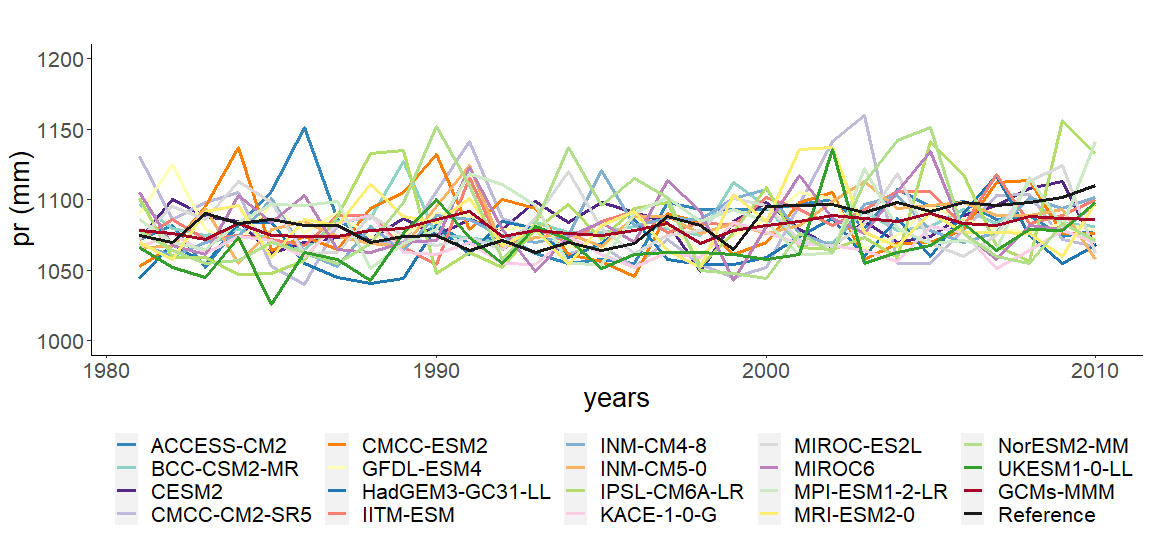


SFig. 11. Globally averaged time series of annual precipitation (pr, mm) from 18 GCMS, multi-model mean (MMM) of GCMs (MMM-GCMs) and the reference precipitation (i.e, MSWEP) for 1981-2010.


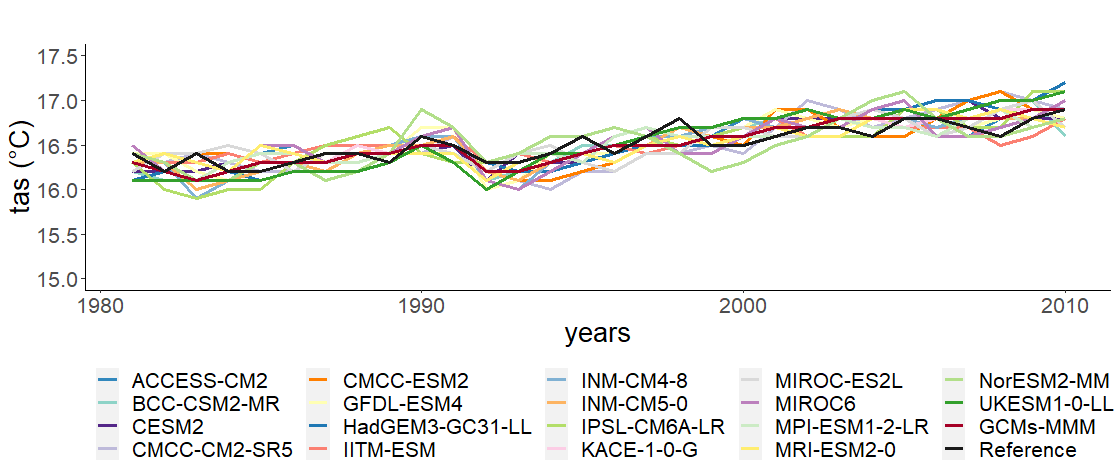


SFig. 12. Globally averaged time series of annual average temperature (tas, °C) from 18 GCMS, multi-model mean (MMM) of GCMs (MMM-GCMs) and the reference temperature (i.e, MSWX) for 1981-2010.


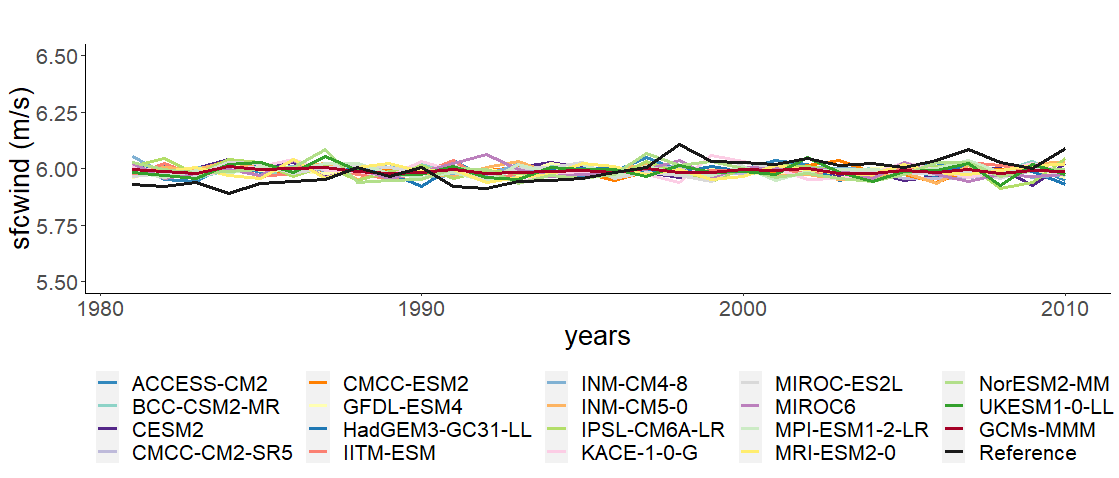


SFig. 13. Globally averaged time series of annual average wind speed (sfcWind, m/s) from 18 GCMS, multi-model mean (MMM) of GCMs (MMM-GCMs) and the reference wind speed (i.e, MSWX) for 1981-2010.


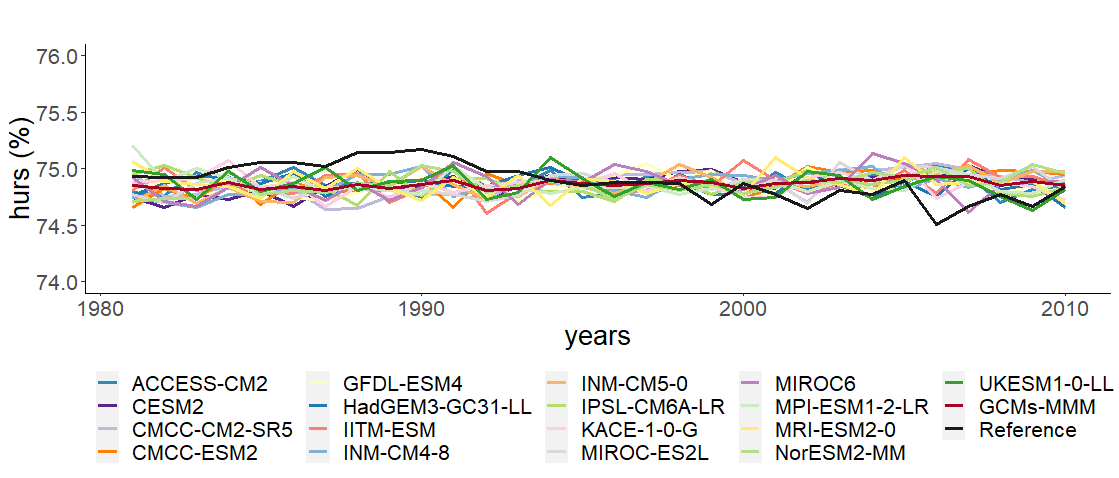


SFig. 14. Globally averaged time series of annual average relative humidity (hurs, %) from 17 GCMS, multi-model mean (MMM) of GCMs (MMM-GCMs) and the reference relative humidity (i.e, MSWX) for 1981-2010.


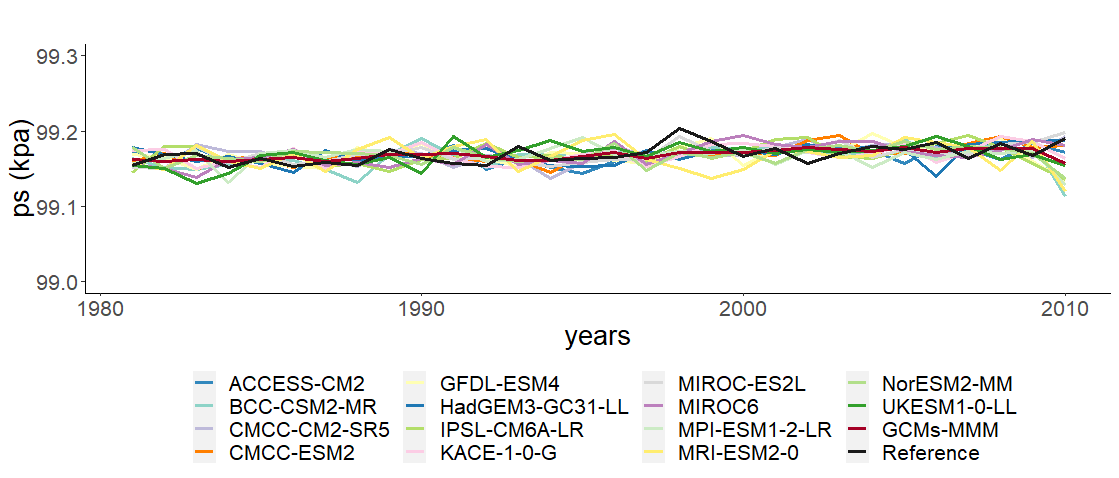


SFig. 15. Globally averaged time series of annual average air-pressure (ps, KPa) from 17 GCMS, multi-model mean (MMM) of GCMs (MMM-GCMs) and the reference air-pressure (i.e, MSWX) for 1981-2010.
